# Primary Hyperparathyroidism: A Common Condition With an Uncommon Location

**DOI:** 10.7759/cureus.76244

**Published:** 2024-12-23

**Authors:** Carolina Monteiro Antunes, Maria Leonor Guia Lopes, Francisco Sousa Santos, Sequeira Duarte

**Affiliations:** 1 Endocrinology Department, Hospital de Egas Moniz - Centro Hospitalar de Lisboa Ocidental, Lisbon, PRT

**Keywords:** ectopic hyperparathyroidism, ectopic parathyroid adenoma, hypercalcemia, parathyroidectomy, primary hyperthyroidism

## Abstract

Primary hyperparathyroidism (PHPT) is a prevalent clinical condition characterized by an inappropriate secretion of parathyroid hormone (PTH). It is most often caused by one or more parathyroid adenomas, which can, in rare cases, be ectopically located. Ectopic adenomas can pose a diagnostic challenge, lead to treatment delay, and be a common cause of recurrent hypercalcemia after parathyroidectomy. We present the case of a 73-year-old woman referred to our Endocrinology Department for hypercalcemia, with initial blood tests confirming primary hyperparathyroidism. Following a negative cervical ultrasound, a parathyroid sestamibi scan was performed, which identified an MIBI (technetium (Tc)-99m methoxyisobutylisonitrile)-avid focus in the midline posterior cervical region suggestive of an ectopic parathyroid adenoma. Subsequently, a four-dimensional neck CT scan revealed a retro-esophageal nodular lesion. The diagnosis was confirmed through esophageal endoscopic ultrasound-guided fine needle aspiration. The patient underwent minimally invasive parathyroidectomy with a significant intraoperative decrease in PTH levels and a postoperative normalization of calcium levels. Six months after surgery the patient shows no signs of recurrence. This clinical case highlights the importance of a thorough diagnostic workup and the use of multiple imaging modalities to accurately locate parathyroid adenomas. This approach helps prevent incorrect surgical procedures and improves treatment outcomes.

## Introduction

Primary hyperparathyroidism (PHPT) is a disorder characterized by hypercalcemia due to an excessive secretion of parathyroid hormone (PTH). PHPT is the most common cause of hypercalcemia, affecting up to seven in 1000 persons and being more prevalent in women in the fifth and sixth decades of life [1-3].

PHPT is more commonly caused by an isolated parathyroid adenoma or parathyroid hyperplasia, but in rare cases may be due to parathyroid carcinoma [2,3]. A small portion of parathyroid adenomas can be ectopically located, due to abnormal migration of the parathyroid glands during embryogenesis. The most frequent locations of ectopic parathyroid adenomas are the tracheoesophageal groove and the retroesophageal area but can also be found in other locations such as intrathyroidal or in the mediastinum [2-5]. In this setting, the adenoma tends to be larger, associated with higher PTH and calcium levels and significantly worse bone disease [4,6]. Ectopic adenomas can pose a diagnostic challenge because they can be overlooked in initial imaging evaluations [7]. Persistent or recurrent hypercalcemia after parathyroidectomy should raise concern for the possibility of an ectopic parathyroid adenoma. If left untreated, PHPT can lead to serious complications such as osteoporosis, kidney stones, and cardiovascular disease, which makes prompt diagnosis and treatment essential. A thorough preoperative investigation, which may require multiple imaging exams, is crucial in such circumstances to avoid unsuccessful surgical procedures.

We report a case of primary hyperparathyroidism due to an ectopic parathyroid adenoma, highlighting the importance of a comprehensive diagnostic workup for accurate localization and successful surgical treatment.

## Case presentation

A 73-year-old woman was referred to our Endocrinology Department due to hypercalcemia noted in a routine blood workup requested in Primary Care. She had a relevant medical history of nephrolithiasis, type 2 diabetes, hypertension, and primary hypothyroidism. Patient medication included levothyroxine, metformin, losartan, and atenolol. She had no history of lithium or bisphosphonates intake and there was no relevant family history. She was asymptomatic and denied any previous fractures. Her physical examination was unremarkable.

Initial evaluation revealed a slight elevation of calcium levels (total calcium 10.8 mg/dL and ionized calcium 5.7 mg/dL) along with a markedly high PTH level (170 pg/mL) suggesting hyperparathyroidism (Table 1).

**Table 1:** Blood and urine analysis.

Laboratory test	Result	Reference range
Hemoglobin (g/dL)	13.5	12.0-15.0
Creatinine (mg/dL)	1.01	0.50-0.90
Glomerular filtration rate (mL/min/1.73m^2^)	55	> 60
Alkaline phosphatase (U/L)	221	35-104
Total calcium (mg/dL)	10.8	8.8-10.2
Ionized calcium(mg/dL)	5.7	4.5-5.3
Phosphate (mg/dL)	3.0	2.5-4.5
Parathyroid hormone (pg/mL)	170	15-65
25-Hydroxyvitamin D (nmol/L)	70	70-250
Creatinine U24h (mg/24h)	603	600-1500
Calcium U24h (mg/24h)	215	100-300

Renal ultrasound evidenced that bilateral lithiasis and dual-energy x-ray absorptiometry (DXA) were compatible with osteoporosis with a T-score of -3.2 at L3-L4 and -2.7 at the femoral neck. The patient was started on vitamin D supplementation with cholecalciferol 1800 IU/day and osteoporosis treatment with alendronic acid 70 mg/week.

**Figure 1:**
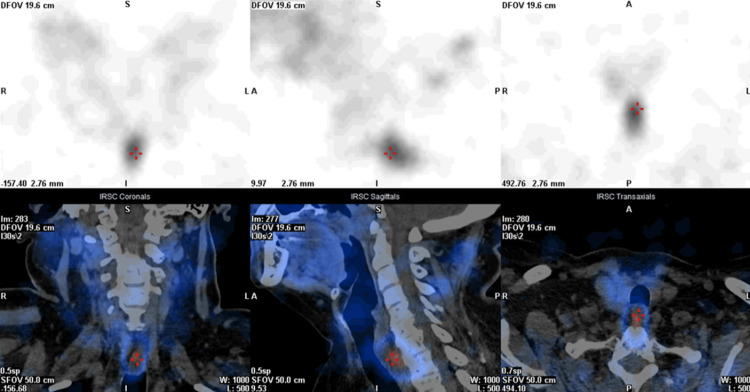
99mTc-sestamibi SPECT/CT indicating a MIBI-avid focus in the midline posterior cervical region (red crosshairs) suggestive of an ectopic parathyroid adenoma. MIBi: Tc-99m methoxyisobutylisonitrile; Tc: technetium; SPECT: single-photon emission computed tomography

Considering the established biochemical diagnosis of PHPT with osteoporosis and nephrolithiasis, surgical intervention was considered to prevent further complications. The follow-up investigation included imaging exams to identify a possible parathyroid adenoma. We started by performing a cervical ultrasound, which showed an intra-thyroidal nodule of the left lobe with 7 mm, with no suggestive enlarged parathyroid glands. Concomitantly, a parathyroid sestamibi single-photon emission computed tomography-computed tomography (SPECT/CT) scan identified a MIBI (technetium (Tc)-99m methoxyisobutylisonitrile)-avid focus in the midline posterior cervical region, probably corresponding to the left inferior parathyroid gland (Figure 1).

**Figure 2:**
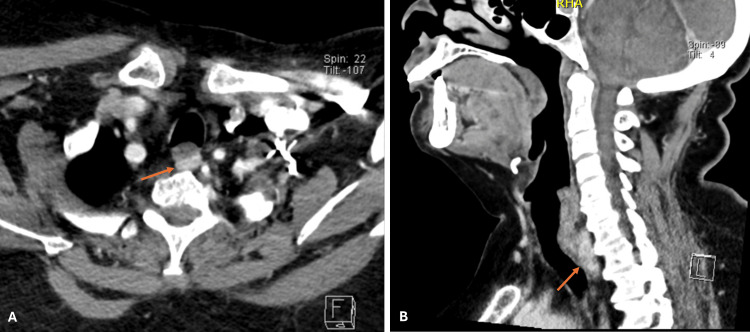
Preoperative four-dimensional neck CT scan in axial (panel A) and sagittal views (panel B) showed a hyper vascular retro-esophageal nodular lesion measuring 31x14x7 mm (orange arrow).

A four-dimensional neck CT scan was then performed and confirmed the presence of a retro-esophageal nodular lesion measuring 31x14x7 mm, which suggested an ectopic parathyroid adenoma (Figure 2).

To confirm the diagnostic hypothesis, an upper digestive endoscopy was performed and, at 20 cm from the incisors, a 30x5 mm nodule was found posteriorly to the esophagus. An ultrasound-guided fine needle aspiration (FNA) was carried out and the cytological examination confirmed a parathyroid origin.

The patient underwent minimally invasive parathyroidectomy, with a significant intraoperative decrease of PTH after removal of the adenoma. Following surgery, the calcium levels decreased to normal values. The Pathology report confirmed that the nodule was a parathyroid adenoma measuring 29x17x10 mm and weighing 2000 mg. Six months after surgery the patient remains asymptomatic with normal calcium levels (9.5 mg/dL) and a slightly elevated PTH (75 pg/mL).

## Discussion

Ectopic hyperparathyroidism is responsible for nearly 10% of PHPT cases [4]. The most common locations are the tracheoesophageal groove and the retroesophageal area, with reported location prevalences differing between series [2,4,5]. It should be suspected when there is a biochemical, established PHPT diagnosis with inconclusive or discordant preoperative imaging exams, or when parathyroid surgery is unsuccessful [7].

To minimize neck exploration and subsequent surgical complications, correct localization of the parathyroid adenoma is crucial. Therefore, it is recommended to have at least two concordant imaging exams prior to the surgical procedure. A proposed algorithm for initial imaging exams includes a neck ultrasound combined with a 99m Tc-sestamibi [8]. In spite of its accessibility, ultrasound is an operator-dependent method and can have confounding factors such as concomitant thyroid nodular disease. It also has a low sensitivity for the identification of retroesophageal glands [5,9]. A four-dimensional CT scan has a higher sensitivity for smaller lesions in comparison to scintigraphy and is also a useful method for the identification of lesions in the retroesophageal and mediastinal spaces [7,10]. Additional investigation includes ultrasound-guided FNA cytology, but ordinary cytology analysis may be insufficient for diagnosis. Measurement of PTH in the aspirate and the use of immunohistochemical markers like thyroid transcription factor 1 (thyroid-specific) and chromogranin A (parathyroid indicator) may be particularly useful to differentiate a parathyroid adenoma from lymphatic or thyroid tissue in challenging cases [7,11].

In the current case, given the retroesophageal location of the parathyroid adenoma, it was not identified on the neck ultrasound. Moreover, the coexistence of thyroid nodules could have been a confounding factor if not correlated with the 99m Tc-sestamibi, as an intrathyroidal ectopic location could have been thought. Since the results of the imaging exams were discordant, a four-dimensional neck CT was ordered, which confirmed the presence of a nodule in a retroesophageal location, corresponding to the location proposed by the scintigraphy. The CT scan allowed an accurate identification of the nodule, in concordance with the scintigraphy. Despite having two concordant imaging exams, due to the location of the nodule, we decided to perform an ultrasound-guided FNA to confirm the diagnosis and prevent a potentially unsuccessful surgical procedure. After confirmation that the nodule was indeed an ectopic parathyroid adenoma, the patient was treated with a minimally invasive parathyroidectomy.

Ectopic parathyroid adenomas are associated with higher serum PTH and calcium levels, larger tumors, and a higher incidence of severe hyperparathyroidism-related bone disease (osteitis fibrosa cystica) [4]. Contrary to the majority of the literature, our patient had only a slight elevation of calcium levels (maximum calcium of 10.8mg/dL) and was asymptomatic with an unremarkable physical exam. The resected adenoma was still heavier than those commonly found in orthotopic locations, which have been reported to weigh 553.7 ± 520.5 mg [12].

Ectopic parathyroid adenomas are a common cause of unsuccessful surgical procedures and recurrent PHPT due to an incorrect location of the disease, with most recurrences happening in the first six months after surgery [7]. Methodical preoperative imaging investigation is paramount for a successful surgical treatment. Our patient had a normalization of the calcium levels postoperatively and these have remained normal after six months of follow-up. Elevations of PTH with normal calcium levels can occur in the postoperative period and are usually transitory, not necessarily indicating a recurrence of the PHPT [13]. The patient maintains regular follow-ups with calcium and PTH measurements.

## Conclusions

This clinical case underscores the importance of a multidisciplinary approach in the evaluation and management of PHPT, particularly in cases involving suspected ectopic adenomas. A comprehensive diagnostic workup, incorporating multiple imaging modalities, is crucial to achieving an accurate diagnosis. This approach enhances treatment outcomes and reduces the risk of disease recurrence.
